# Supplementary material for: Intervention Strategies to Overcome HPV Vaccine Hesitancy Among Hispanic Immigrants in the USA: A Video-Based Approach
Source: Vaccines (Basel). 2025 May 28;13(6):574. doi: 10.3390/vaccines13060574 (PMC12197449; doi:10.3390/vaccines13060574)
Supplement: Supplementary file 1 [file vaccines-13-00574-s001.zip › vaccines-3625016-supplementary.pdf]

Supplemental Materials.

Table S1. Video content, format and information on presenter

Video Title (Time)	Video Content	Presenter	Video Format
Summary Video (5:14)	• Viruses infect cells and use them to reproduce.<br>• HPV can cause warts and other signs or remain dormant with no symptoms.<br>• HPV causes 90% of cervical cancers, 70% of throat cancers, and 90% of genital warts.<br>• HPV is transmitted through sexual intercourse and skin-to-skin contact.<br>• The HPV vaccine contains parts of the virus (proteins) but no genetic material.<br>• The vaccine helps the immune system recognize and fight the virus.<br>• The body's immune system is like a castle, and the guards (immune system) fight invaders (virus).<br>• HPV vaccine decreases infections by 88% in adolescents and 81% in young adult women.<br>• The vaccine underwent rigorous testing and is proven safe by the FDA.	A male Biology graduate student who is a native Spanish speaker.<br><br>A male medical Doctor who is a non-native Spanish speaker.<br><br>A male microbiology university professor who is a non-native Spanish speaker.<br><br>A female pre-dental undergraduate student who is a native Spanish speaker.	Whiteboard animation and real individuals talking
What is HPV? (2:24)	• HPV is a virus that inserts its genetic material into cells and reprograms them.<br>• It can cause warts or remain dormant without symptoms for months or years.<br>• The virus can eventually cause cells to reproduce uncontrollably, leading to cancer.<br>• HPV infections are a permanent risk factor for cancer.<br>• Vaccination prevents initial HPV infection, reducing the future risk of cancer.	A male Biology graduate student who is a native Spanish speaker	White board animation
How Do I Prevent HPV? (3:01)	• The HPV vaccine is the best way to protect against HPV.<br>• The vaccine contains proteins from the virus but no genetic material.<br>• The vaccine helps the immune system recognize and fight the virus.	A male microbiology university professor who is a non-native Spanish speaker.<br><br>A female pre-dental undergraduate student	White board animation and real individuals talking

	• The immune system produces antibodies to defend against future infections.<br>• Vaccination prepares the immune system like guards defending a castle.<br>• The vaccine teaches the immune system to recognize and destroy the virus.	who is a non-native Spanish speaker.	
Where does the vaccine come from? (3:06)	• The HPV vaccine was rigorously tested and approved by the FDA.<br>• Extensive tracking over 15 years shows the vaccine is safe.<br>• The vaccine has been proven effective in preventing HPV infections and related conditions.<br>• Side effects are usually mild and temporary (pain, fever, dizziness).<br>• HPV infections and precancerous cervical lesions have decreased by 88% in adolescents.<br>• The vaccine offers long-term protection, lasting over 12 years with robust immunity.	A male Biology graduate student who is a native Spanish speaker	White board animation
At what age should I vaccinate my children against HPV? (2:08)	• The vaccine is recommended for ages 9-45, with the best age being 11-13.<br>• Vaccinating before adolescence ensures the immune system is prepared before exposure.<br>• Vaccination is advised before children explore physical intimacy.<br>• The vaccine does not encourage sexual behavior, as research shows no link.<br>• Administering the vaccine at 12-13 years aligns with other routine vaccinations.	A male Biology graduate student who is a native Spanish speaker	White board animation
Are there side effects of getting vaccinated? (3:09)	• The HPV vaccine is very safe and has minimal side effects.<br>• Common side effects: pain, redness, swelling at the injection site, fever, dizziness, nausea, and fatigue.<br>• Side effects are usually resolved within one to two days.<br>• The vaccine has no long-term side effects and does not affect fertility.<br>• The vaccine helps prevent cancer-related fertility issues caused by HPV.	A male Biology graduate student who is a non-native Spanish speaker	White board animation

	• A 99% effectiveness rate in preventing persistent HPV infections and related conditions.		
Testimonials (7:54)	• Parents chose to vaccinate their children to prevent HPV and related diseases.<br>• Vaccination is a preventive measure, not an endorsement of sexual behavior.<br>• Vaccinated children, particularly girls and boys, are protected against future health issues.<br>• HPV vaccine is effective and high in protection against cervical cancer.<br>• Vaccination should occur before adolescence, ideally before age 15, to prevent infection.<br>• Vaccines prevent future health risks, and preparation is key as parents cannot control future decisions.<br>• Parents emphasized the importance of vaccination to protect children from future health problems.<br>• Parents discussed the safety of the vaccine and its minimal side effects.<br>• No serious reactions have been associated with the HPV vaccine	Three women who are all native Spanish speakers	Real individuals talking

Table S2. Video Title, Duration and YouTube link

Video Title	Duration	Link
Summary video	5:14	https://www.youtube.com/watch?v=uEKLu7JuZz0
What is HPV?	2:25	https://www.youtube.com/watch?v=vCRaGdHcLQA
How Do I Prevent HPV?	3:02	https://www.youtube.com/watch?v=NVXGu_y86kw
Where did the HPV Vaccine Come From?	3:06	https://www.youtube.com/watch?v=hKYOlr50qG0
At What Age Should Children Be Vaccinated?	2:09	https://www.youtube.com/watch?v=vhIKLkzXFXA
Are There Any Side Effects to Getting Vaccinated?	3:10	https://www.youtube.com/watch?v=phwWZz-73jU

Testimonials	7:54	https://www.youtube.com/watch?v=fMKiKDVUq1I

Table S3: Confirmatory Factor Analysis

**Latent Factor**	**RMSEA**	**CFI**	**TLI**	**SRMR**
General Vaccine Attitudes	0.000	1.000	1.000	0.007
HPV Vaccine Uptake	0.034	0.998	0.995	0.007
HPV Vaccine Attitude	0.032	0.998	0.995	0.006
Combined General Vaccine Attitude and HPV Vaccine Attitude	0.030	0.994	0.991	0.017
HPV Knowledge	0.068	0.989	0.968	0.016
English Proficiency	0.000	1.000	1.000	0.004
Openness/Comfort/Fear	0.059	0.985	0.954	0.023
Care Level	0.037	0.985	0.991	0.011
Video Feedback	0.062	0.964	0.954	0.027
Combined Pre-Survey Factors	0.042	0.962	0.956	0.054

Table S4: Demographics of Participants

**Characteristic**		**Total N (%)**
Gender	Male	418 (35.2%)
	Female	757 (63.7%)
	Missing	13 (1.1%)
Age	Less than 18	0 (0%)
	18-25	163 (13.7%)
	26-35	390 (32.8%)
	36-45	347 (29.2%)
	46-55	135 (11.4%)
	Over 55	152 (12.8%)
	Missing	1 (0.1%)
Race	Native American or Alaska Native	8 (0.7%)
	Asian	1 (0.1%)
	Black	9 (0.8%)
	Hispanic/Latino	880 (74.1%)
	White	106 (8.9%)
	Other	10 (0.8%)
	Mixed Race	173 (14.6%)
	Missing	1 (0.1%)
Latin Descent	Not of Hispanic, Latino, or Spanish origin	0 (0%)
	Mexican, Mexican American, Chicano	496 (41.8%)
	Puerto Rican	153 (12.9%)
	Cuban	199 (16.8%)
	Another Hispanic, Latino, or Spanish origin (Salvadorian, Dominican, etc.)	339 (28.5%)
	Missing	1 (0.1%)

Length of Residency in USA	Less Than One Year	64 (5.4%)
	1-5 Years	255 (21.5%)
	More Than 5 Years	867 (73%)
Income	Less than $5,000	58 (4.9%)
	$5,000-15,000	85 (7.2%)
	$16,000-25,000	124 (10.4%)
	$26,000-$35,000	123 (10.4%)
	$36,000-$45,000	106 (8.9%)
	$45,000-$60,000	185 (15.6%)
	$61,000-$75,000	151 (12.7%)
	$76,000-$100,000	187 (15.7%)
	Over $100,000	163 (13.7%)
	Missing	6 (0.5%)
Children	Yes	1187 (99.9%)
	No	1 (0.1%)
	Missing	1 (0.1%)
Number of Children	1	86 (7.2%)
	2	403 (33.9%)
	3	275 (23.1%)
	4	84 (7.1%)
	5	234 (19.7%)
	5+	96 (8.1%)
	Missing	10 (0.8%)
Marital Status	Single	148 (12.5%)
	Partnered	190 (16%)
	Married	698 (58.8%)
	Divorced	116 (9.8%)
	Widow/Widower	25 (2.1%)
	Missing	11 (0.9%)
Most of My Family Lives...	In the United States	716 (60.3%)
	In my home country	391 (32.9%)
	In another country besides the United States or my home country	76 (6.4%)
	Missing	5 (0.4%)
Education	Have not finished high school (1)	86 (7.2%)
	Finished high school (2)	403 (33.9%)
	Some college or vocational certificate (3)	275 (23.1%)
	Associate’s degree (4)	84 (7.1%)
	Bachelor’s degree (5)	234 (19.7%)
	Advanced degree (Masters, MD, DDS, PhD, etc.) (6)	96 (8.1%)
	Missing	10 (0.8%)

Table S5: Resources Used in Video Preparation

Resource Name	Version	Purpose	Developer/Publisher	URL
**WavePad**	19.36	Audio editing	NCH Software	https://www.nch.com.au/wavepad/
**VideoScribe**	3.12	Video animation creation	Sparkol Ltd	https://www.videoscribe.co/
**iMovie (macOS)**	10.38	Video editing	Apple Inc.	https://www.apple.com/imovie/

Video Transcripts/ English

Summary video

Viruses are microbes that infect your cells and use them to reproduce. When one first enters, it introduces genetic material that reprograms the infected patient's cells. When the HPV virus first infects a cell and inserts its genetic material, it can immediately start making copies of itself, causing warts and other physical signs of disease. But it can also remain dormant or show no symptoms. It is impossible to know who will develop health problems from an HPV infection, and a person can have it for years before developing symptoms.

It is a disease-causing, even life-threatening, virus that causes more than 90% of cervical cancers, 70% of all throat cancers and 90% of all genital warts. HPV can be transmitted through sexual intercourse, as well as through skin-to-skin contact with a person infected with the virus. People become infected without knowing it and can then pass it on to a partner or spouse.

HPV vaccines are a necessary step to prevent such tragedies. A vaccine is something that is injected into us and contains parts of a virus. In this case, those parts are called proteins. It is important to recognize that there is no genetic material in this vaccine. What it does is help our immune system recognize the virus so that the next time we are infected, we don't have symptoms. Our immune system will be able to fight and defend against it.

Your body is like a castle, and your immune system is like the guards that defend it. The vaccine allows the guards to identify the intruder before it reaches the castle and be ready to defend it. By creating a mimic

of the virus, researchers can cause the body to produce and store the antibody without having to get sick. This is how we prepare the guards to defend our castle.

In adolescents, infections with the HPV types responsible for most HPV-related cancers and genital warts have decreased by 88%. In young adult women, infections with the same HPV types have decreased by 81%. These remarkable statistics highlight the effectiveness of HPV vaccination in preventing serious health problems. The protection offered by HPV vaccines lasts for a long period of time.

The HPV vaccine underwent rigorous safety testing before being approved by the US Food and Drug Administration (FDA). Extensive monitoring and research conducted over 15 years in vaccination programmes consistently demonstrate that HPV vaccination is safe.

The vaccine is available between the ages of 9 and 45, but most doctors recommend that it be given between the ages of 11 and 13. Doctors believe it is necessary to receive the vaccine at this young age because the vaccine prevents infection. Because it is sexually transmitted, children should receive it before they have a chance to explore intimacy.

Some may worry that administering the vaccine will encourage sexual behaviour in children. However, the Centers for Disease Control and Prevention (CDC) reports that research studies have shown that this is not the case.

HPV vaccines, like any medicine, can have side effects. Most of these side effects are mild and usually get better within a day or two. The following are some common side effects associated with HPV vaccines: pain, redness or swelling at the injection site; fever; dizziness or fainting; nausea; headache or fatigue; and muscle pain.

Video 1-What is HPV?

The main goal of any parent is to protect their children. We make sure that they have the appropriate clothing when it's cold, they have healthy food for lunch, and they have good friends at school. But with so much conflicting information and messages out there, it's easy to get confused about what can help or hurt our children, especially with issues we don't usually have much information about, such as vaccines.

Our goal as researchers, scientists, and teachers is to help you receive the right information to make an informed decision about whether the HPV vaccine will benefit the well-being of your children. The human papillomavirus, or HPV, is a unique virus. Viruses are microbes that infect your cells and use them to reproduce. When a virus enters your body for the first time, it introduces genetic material that reprograms the infected patient's cells.

HPV is a special type of virus. When the HPV virus infects a cell for the first time and inserts its genetic material, it can immediately start making copies of itself, causing warts and other physical signs of illness. However, it can also remain dormant, not showing symptoms for months, years, or even decades, while its genetic material is inserted directly into our own cells.

Over time, the genetic material of the virus can be activated and start creating more copies using the cellular machinery, destroying the cell and spreading to other neighboring cells. The insertion of viral genetic material into cells can cause other problems, such as interrupting important regulatory genes. This can make the cell reproduce too quickly and, therefore, provoke cancer in the people it infects.

Once the genetic material has been inserted, it is impossible to remove it from the cell, becoming a permanent risk factor for cancer later in life. Because of this, we use a vaccine that prevents the virus from being contracted in the first place.

Video 2-How do I prevent HPV?

The best way to protect yourself from HPV is through the vaccine against this virus. But what exactly is the HPV vaccine? My name is Brett Picket, and I have a PhD in microbiology. I am now a teacher and scientist at Brigham Young University in the United States. A vaccine is something that is injected into us

and contains parts of a virus. In this case, the parts of the virus are called proteins. It is important to recognize that in this vaccine, there is no genetic material.

The purpose of the vaccine is to help our immune system recognize the virus so that the next time we are exposed, we don't experience symptoms or illness. Essentially, the vaccine trains our immune system to fight and defend against the virus. When a person receives the vaccine, they are protected from future infections and it helps their body avoid getting sick when exposed to the virus in the future.

Think of your body as a castle, and your immune system as the guards defending it. Vaccination allows the guards to identify the intruder before it reaches the castle and prepares them to defend it. This is a simplified explanation of how an immune reaction works when the body is exposed to a pathogen, such as a virus or bacterium. The immune system creates an antibody that binds to the pathogen, marking it so it can be destroyed. Without antibodies, the virus would be free to replicate and take control.

Once the body has identified the pathogen through the appropriate antibody, the immune system can eliminate the virus from the body. Additionally, the immune system stores memory of the antibody indefinitely. This means that if it encounters the same virus again, it can quickly attack and destroy it. This process is called immunity. By creating a virus imitation, researchers can trigger the body to produce and store the antibody without requiring the individual to become sick. In this way, the vaccine prepares the guards to defend our body.

Video 3- Where did the vaccine come from?

The HPV vaccine was subjected to rigorous safety testing before receiving approval from the U.S. Food and Drug Administration (FDA). Exhaustive tracking and investigations conducted over 15 years in vaccination programs have systematically demonstrated that vaccination against HPV is safe. This vaccine underwent rigorous clinical testing to prove its safety and efficacy, as is the case with all vaccines approved by both the Centers for Disease Control and Prevention (CDC) and the FDA. Both agencies closely monitor the safety of HPV vaccines, and if a safety concern arises, it is promptly reported to health authorities, healthcare professionals, and the public.

Like any other medicine, HPV vaccines may have side effects. Most of these side effects are mild and typically improve within one or two days. Common side effects associated with HPV vaccines include pain, redness, or swelling at the injection site; fever; dizziness or fainting; nausea; headache or fatigue; and muscle or joint pain. It is important to remember that these side effects are usually temporary.

Since 2006, when HPV vaccines were first introduced in the United States, there has been a significant reduction in HPV infections and related conditions. Rates of precancerous cervical lesions and genital warts have decreased by 88% among adolescents and young adult women. Additionally, HPV infections among vaccinated women have decreased by 81%, and vaccinated women have experienced a 40% reduction in cervical precancer rates.

These remarkable statistics highlight the effectiveness of HPV vaccination in preventing serious health problems. Regular vaccinations and continuous monitoring are crucial to maintaining this positive trend. The protection offered by HPV vaccination lasts for a prolonged period. People who received these vaccines have been closely monitored for at least 12 years, and their immunity against HPV has remained robust with no signs of decline.

Video 4- At what age is it recommended?

Many people wonder at what age their children should be vaccinated and if it is dangerous to do it too soon. Clinical trials have been conducted in children as young as 9 years old, which is the earliest age that a doctor would commonly recommend vaccination. The vaccine is available for individuals between 9 and 45 years of age, but most doctors recommend administering it between the ages of 11 and 13. Doctors consider it necessary to vaccinate at this early age because the vaccine prevents infection; it does not help

individuals who are already infected. Since HPV is transmitted through sexual contact, children should receive the vaccine before they have the possibility of exploring physical intimacy.

Administering the vaccine before puberty ensures that the immune system is prepared before any potential exposure to the virus. Just as a castle needs guards before invaders arrive, your child needs to be vaccinated against HPV before there is any chance of infection. Some may worry that administering the vaccine could encourage sexual behavior in children and adolescents, but research studies conducted by the Centers for Disease Control and Prevention (CDC) have shown that this is not the case. Children are no more likely to become sexually active after receiving the vaccine.

In addition, children between 12 and 13 years of age typically receive many other vaccines, so administering the HPV vaccine at this time can reduce the total number of doctor visits, which can save money. For this reason, pediatricians recommend starting the vaccination process as soon as possible.

Video 5- Are there side effects?

You will naturally want to know how this vaccine will affect your child. Is it really safe? What will it do to my child? The HPV vaccine is very safe. It is designed to look like HPV so that your immune system can recognize it, but it contains none of the components that make us sick. As we have explained before, getting vaccinated is like giving the guards of a castle the enemy's battle plan. It gives the immune system time to strategize and prepare before an attack. When the attack comes, the immune system is well-prepared to fight the enemy and prevent it from taking over the castle—or, in this case, your body.

Studies have shown that the vaccine has a 99% efficiency in preventing persistent HPV infections, genital warts, and cervical cancer caused by HPV. However, the vaccine does not eliminate the need for regular cervical cancer screening tests, as other factors can contribute to the development of this disease. Your doctor may recommend not vaccinating your child if they suspect a possible allergic reaction, if the child is very ill, or if the individual is pregnant.

Like all vaccines, the HPV vaccine can have some minor but unpleasant side effects, such as pain, redness, and swelling at the injection site, mild fever, and aches. Between 10% and 13% of individuals who received the vaccine reported having a fever of approximately 100°F (38°C). These side effects are signs that the immune system is beginning to build defenses against HPV, which will protect the individual if they come into contact with the virus later.

There have been no serious reactions associated with the HPV vaccine. Many people experienced the same symptoms as they would with other vaccines. Some people wonder whether getting vaccinated will affect fertility. The HPV vaccine does not cause infertility. In fact, it helps prevent cancers that can affect fertility. HPV can cause cancers of the reproductive organs in both men and women, which could impact fertility in the future. The best way to protect against infertility caused by HPV-associated cancers is to get vaccinated against HPV.

Video 6- Testimonial from Pro-Vaccine Parents

My name is ……., and I have three children. My name is …….., and I have four children. I chose to vaccinate my children for prevention—prevention of different diseases, specifically HPV. I didn't vaccinate all my children, only the girls, and my son. As a mother, we always want to protect our children. If there's any way I can help prevent harm in the future, such as disease, I will do it. One of those ways is to vaccinate them. I like vaccinating my children for the same reason—to prevent any illness or harm that might occur. The HPV vaccine has been thoroughly researched, and its protection rate is very high. I believe it is a good decision.

The vaccine should be administered before adolescence. If I remember correctly, it must be given before the age of 15. At that age, children don't even know what sex is, so it has nothing to do with their sexual relationships. It's simply about prevention, not permission to have sex. It's a way to prevent

something that might happen because we don't know the future. In the end, the decision will be theirs, but it's definitely not permission. As a parent, I am responsible for my children, and I prefer to prevent things that could happen in the future—not that they will happen, but they could.

I think the expectations of a family and how you educate your children cover certain parts of their growth. At the same time, we don't know the decisions they will make in the future. While I cannot make decisions for them, this is no reason not to prepare them. As a mother, it is my job to cover all the bases. Their job is to make good decisions, but sometimes they don't know how to do that.

I am the daughter of a nurse, so I grew up in the medical world. My family has always believed in vaccines. Now, I am married to a chiropractor, and chiropractors by nature don't believe in vaccines. We had to select which vaccines we would use for our children. We chose vaccines that could prevent life-threatening illnesses. The HPV vaccine prevents cervical cancer. If it isn't administered before someone starts having sex, it may be too late because one of the HPV strains could already have infected the individual. Women suffer from this disease, while men may transmit it but don't suffer from it. For this reason, I decided to vaccinate my children when they were about 12 years old.

I don't believe the vaccine conflicts with my religion. It's not about teaching them something or giving them permission to do anything; it's simply about prevention. At the end of the day, everyone has their free will and the right to decide for themselves. A vaccine has nothing to do with religion; it's about protecting them. I obviously teach my children not to have sexual relationships before marriage for many reasons. When considering this vaccine, I initially thought it might not be necessary because we don't teach that lifestyle. However, based my decision on their physical health was my primary reason.

Every vaccine, just like everything we eat or drink, has potential side effects. But if it can prevent cervical cancer, I believe it's worth it. From what I've read and studied, the vaccine prevents one of the most common cancers. Although there are many types, preventing this particular one makes the vaccine worthwhile. Vaccines are approved by the Department of Health only after going through rigorous studies over many years. Once approved, they are deemed safe for public use. The side effects of the vaccine are minimal compared to the risks of acquiring the disease.

I've had family and friends affected by HPV. Even though they believed in abstinence until marriage, their decisions didn't protect them from HPV. They had to undergo operations, and one friend experienced fertility problems due to surgery. When you weigh the risks and options, it's better to prepare for the unknown. We can hope our children make virtuous choices, but we cannot control them. We can only prepare them. I'd rather be safe than regret not making the decision to vaccinate because the choice was left up to me as a parent. Children are not attentive to these things, so the decision falls to us as parents to protect them.

Survey – Spanish

DEMOGRAFÍA

Q1. ¿Es usted de origen hispano, latino o español?

1. No, no soy de origen hispano, latino o español
2. Sí, mexicano, mexicano-americano, chicano
3. Sí, puertorriqueño
4. Sí, cubano
5. Sí, soy de otro origen hispano, latino o español; por ejemplo, salvadoreño, dominicano, colombiano, guatemalteco, español, ecuatoriano, etc.

[Saltar a: Fin del bloque Si ¿Es usted de origen hispano, latino o español? = No, no soy de origen hispano, latino o español].

Q2. ¿Habla español con fluidez?
1. Sí
2. No

[Saltar a: Fin de bloque Si ¿Habla español con fluidez? = No]

Q3. ¿Cuánto tiempo lleva viviendo en Estados Unidos?
1. Menos de un año
2. De 1 a 5 años
3. Más de 5 años

Q4. ¿Tiene hijos?
1. Sí
2. No

[Pase a: Fin del bloque Si tiene hijos = No]

Q5. ¿Cuántos años tienes?
1. Menos de 18 años
2. Entre 18 y 25 años
3. Entre 26 y 35 años
4. Entre 36 y 45 años
5. Entre 46 y 55 años
6. Más de 56 años

[Pasar a: Fin de bloque Si ¿Cuántos años tiene? = Menos de 18 años]

Q6. Raza: Seleccione todas las que correspondan.
1. Indio americano o nativo de Alaska
2. Asiáticos
3. Negros
4. Hispano/Latino
5. Blanco
6. Otros (especifique)

Q7. Sexo
1. Masculino
2. Femenino
3. No binario/otro

Q8. ¿Cuál de las siguientes opciones describe mejor los ingresos anuales de su hogar?
1. Menos de 5.000 $
2. Entre 5.000 y 15.000 $
3. Entre 16.000 y 25.000 dólares
4. Entre 26.000 y 35.000 dólares
5. Entre 36.000 y 45.000 dólares
6. Entre 45.000 y 60.000 dólares
7. Entre 61.000 y 75.000 dólares
8. Entre 76.000 y 100.000 dólares
9. Más de 100.000 dólares

Q9. Estado civil
1. Soltero
2. Pareja/unión libre
3. Casados
4. Divorciado
5. Viuda

Q10. Educación
1. No terminó la enseñanza secundaria
2. Terminó la enseñanza secundaria
3. Algún título universitario o de formación profesional
4. Diplomatura
5. Título de bachillerato (universidad)
6. Titulación superior (máster, medicina, odontología, doctorado, etc.)

Q11. ¿Cuántos hijos tiene?
1. 1
2. 2
3. 3
4. 4
5. 5 o más

Q12. La mayor parte de mi familia vive...
1. En los Estados Unidos
2. En mi país de origen
3. En un país distinto de Estados Unidos o de mi país de origen

ACTITUDES GENERALES ANTE LAS VACUNAS
Indique su grado de acuerdo con cada una de estas afirmaciones.
Escala: (1) Totalmente en desacuerdo
(2) En desacuerdo
(3) Ni de acuerdo ni en desacuerdo
(4) De acuerdo
(5) Totalmente de acuerdo

Q1. Las vacunas son más beneficiosas que perjudiciales.
Q2. Las vacunas son eficaces para prevenir enfermedades.
Q3. Las vacunas se evalúan exhaustivamente para garantizar su seguridad.
Q4. Mis hijos han recibido todas las vacunas recomendadas.

Q5. Los esfuerzos de vacunación han reducido las enfermedades infecciosas en Estados Unidos.

ACEPTACIÓN DE LA VACUNA CONTRA EL VPH
Q1. ¿Se ha vacunado contra el VPH?
1. Sí
2. No

3. No lo sé

Q2. ¿Cuál de las siguientes opciones describe mejor su actitud sobre la administración de la vacuna contra el VPH a sus hijos?

1. No vacunaré a mis hijos
2. Probablemente no vacunaré a mis hijos
3. Estoy pensando en vacunar a mis hijos, pero aún no he tomado una decisión
4. Pienso vacunar a mis hijos
5. Ya he vacunado a mis hijos

Indique su grado de acuerdo con cada una de estas afirmaciones.
Escala: (1) Totalmente en desacuerdo
(2) En desacuerdo
(3) Ni de acuerdo ni en desacuerdo
(4) De acuerdo
(5) Totalmente de acuerdo

Q3. Recomendaría la vacuna contra el VPH a mis vecinos y amigos.
Q4. Animaría a mi familia a vacunarse y a vacunar a sus hijos.
Q5. Estoy a favor de que mi familia se vacune contra el VPH.

ACTITUDES HACIA LA VACUNA DEL VPH.
Indique su grado de acuerdo con cada una de estas afirmaciones.
Escala: (1) Totalmente en desacuerdo
(2) En desacuerdo
(3) Ni de acuerdo ni en desacuerdo
(4) De acuerdo
(5) Totalmente de acuerdo

Q1. Creo que es seguro administrar la vacuna contra el VPH a mis hijos.
Q2. Creo que la vacuna contra el VPH es una medida preventiva necesaria para mis hijos.
Q3. Creo que la vacuna está diseñada para proteger a mis hijos.
Q4. Creo que la vacuna es más útil que perjudicial.

CONOCIMIENTO DEL VPH
Indique su grado de acuerdo con cada una de estas afirmaciones.
Escala: (1) Totalmente en desacuerdo
(2) En desacuerdo
(3) Ni de acuerdo ni en desacuerdo
(4) De acuerdo
(5) Totalmente de acuerdo

Q1. El VPH es una infección potencialmente mortal.
Q2. La infección por VPH puede causar graves sufrimientos físicos.
Q3. Sólo una pequeña minoría de personas contraerá el VPH a lo largo de su vida.
Q4. El VPH causa una cantidad considerable de cáncer en mujeres y hombres.

APERTURA/COMODIDAD/MIEDO

Q1. Me gustaría obtener más información sobre la vacuna contra el VPH.

1. Totalmente en desacuerdo
2. En desacuerdo
3. Ni de acuerdo ni en desacuerdo
4. De acuerdo
5. Totalmente de acuerdo

¿Cuál de las siguientes opciones describe mejor su actitud ante la vacunación contra…. Ya he vacunado a mis hijos[JJ1] .

Q2. Podría plantearme vacunar a mis hijos si obtuviera más información al respecto.

1. Totalmente en desacuerdo
2. En desacuerdo
3. Ni de acuerdo ni en desacuerdo
4. De acuerdo
5. Totalmente de acuerdo

Q3. Tengo miedo de la vacuna

1. Totalmente en desacuerdo
2. En desacuerdo
3. Ni de acuerdo ni en desacuerdo
4. De acuerdo
5. Muy de acuerdo

Q4. Me siento cómodo decidiendo sobre la vacuna contra el VPH

1. Totalmente en desacuerdo
2. En desacuerdo
3. Ni de acuerdo ni en desacuerdo
4. De acuerdo
5. Totalmente de acuerdo

ACTITUDES GENERALES ANTE LAS VACUNAS

Indique su grado de acuerdo con cada una de estas afirmaciones.

Escala: (1) Totalmente en desacuerdo
(2) En desacuerdo
(3) Ni de acuerdo ni en desacuerdo
(4) De acuerdo
(5) Totalmente de acuerdo

Q1. Las vacunas son más beneficiosas que perjudiciales.
Q2. Las vacunas son eficaces para prevenir enfermedades.
Q3. Las vacunas se evalúan exhaustivamente para garantizar su seguridad.
Q4. Mis hijos han recibido todas las vacunas recomendadas.
Q5. Los esfuerzos de vacunación han reducido las enfermedades infecciosas en Estados Unidos.

ACEPTACIÓN DE LA VACUNA CONTRA EL VPH

Q1. ¿Se ha vacunado contra el VPH?
1. Sí
2. No
3. No lo sé

Q2. ¿Cuál de las siguientes opciones describe mejor su actitud sobre la administración de la vacuna contra el VPH a sus hijos?
1. No vacunaré a mis hijos
2. Probablemente no vacunaré a mis hijos
3. Estoy pensando en vacunar a mis hijos, pero aún no he tomado una decisión
4. Pienso vacunar a mis hijos
5. Ya he vacunado a mis hijos

Indique su grado de acuerdo con cada una de estas afirmaciones.
Escala: (1) Totalmente en desacuerdo
(2) En desacuerdo
(3) Ni de acuerdo ni en desacuerdo
(4) De acuerdo
(5) Totalmente de acuerdo

Q3. Recomendaría la vacuna contra el VPH a mis vecinos y amigos.
Q4. Animaría a mi familia a vacunarse y a vacunar a sus hijos.
Q5. Estoy a favor de que mi familia se vacune contra el VPH.

ACTITUDES HACIA LA VACUNA DEL VPH.
Indique su grado de acuerdo con cada una de estas afirmaciones.
Escala: (1) Totalmente en desacuerdo
(2) En desacuerdo
(3) Ni de acuerdo ni en desacuerdo
(4) De acuerdo
(5) Totalmente de acuerdo

Q1. Creo que es seguro administrar la vacuna contra el VPH a mis hijos.
Q2. Creo que la vacuna contra el VPH es una medida preventiva necesaria para mis hijos.
Q3. Creo que la vacuna está diseñada para proteger a mis hijos.
Q4. Creo que la vacuna es más útil que perjudicial.

CONOCIMIENTO DEL VPH
Indique su grado de acuerdo con cada una de estas afirmaciones.
Escala: (1) Totalmente en desacuerdo
(2) En desacuerdo
(3) Ni de acuerdo ni en desacuerdo
(4) De acuerdo
(5) Totalmente de acuerdo

Q1. El VPH es una infección potencialmente mortal.
Q2. La infección por VPH puede causar graves sufrimientos físicos.

Q3. Sólo una pequeña minoría de personas contraerá el VPH a lo largo de su vida.
Q4. El VPH causa una cantidad considerable de cáncer en mujeres y hombres.

APERTURA/COMODIDAD/MIEDO
Q1. Me gustaría obtener más información sobre la vacuna contra el VPH.
1. Totalmente en desacuerdo
2. En desacuerdo
3. Ni de acuerdo ni en desacuerdo
4. De acuerdo
5. Totalmente de acuerdo

¿Cuál de las siguientes opciones describe mejor su actitud respecto a la vacunación contra.... Ya he vacunado a mis hijos[JJ1] .

Q2. Podría plantearme vacunar a mis hijos si obtuviera más información al respecto.
1. Totalmente en desacuerdo
2. En desacuerdo
3. Ni de acuerdo ni en desacuerdo
4. De acuerdo
5. Totalmente de acuerdo

Q3. Tengo miedo de la vacuna
1. Totalmente en desacuerdo
2. En desacuerdo
3. Ni de acuerdo ni en desacuerdo
4. De acuerdo
5. Muy de acuerdo

Q4. Me siento cómodo decidiendo sobre la vacuna contra el VPH
1. Totalmente en desacuerdo
2. En desacuerdo
3. Ni de acuerdo ni en desacuerdo
4. De acuerdo
5. Totalmente de acuerdo

Survey – English

DEMOGRAPHICS
Q1. Are you of Hispanic, Latino or Spanish origin?
1. No, I am not of Hispanic, Latino or Spanish origin
2. Yes, Mexican, Mexican American, Chicano
3. Yes, Puerto Rican
4. Yes, Cuban
5. Yes, I am of other Hispanic, Latino or Spanish origin, e.g. Salvadoran, Dominican, Colombian, Guatemalan, Spanish, Ecuadorian, etc.

[Skip To: End of Block If Are you of Hispanic, Latino or Spanish origin? = No, I am not of Hispanic, Latino or Spanish origin.]

Q2. Do you speak Spanish fluently?
1. Yes
2. No

[Skip To: End of Block If Do you speak Spanish fluently? = No]

Q3. How long have you been living in the United States?
1. Less than one year
2. 1 - 5 years
3. More than 5 years

Q4. Do you have children?
1. Yes
2. No

[Skip To: End of Block If Do you have children = No]

Q5. How old are you?
1. Less than 18 years old
2. Between 18 and 25 years old
3. Between 26 and 35 years old
4. Between 36 and 45 years old
5. Between 46 and 55 years old
6. Over 56 years old

[Skip To: End of Block If How old are you? = Under 18 years of age]

Q6. Race: Select all that apply.
1. American Indian or Alaska Native
2. Asian
3. Black
4. Hispanic/Latino
5. White
6. Other (specify)

Q7. Sex
1. Male
2. Female
3. Non-binary/Other

Q8. Which of the following best describes your annual household income?
1. Less than $5,000
2. Between $5,000 and 15,000
3. Between $16,000 and 25,000
4. Between $26,000 and $35,000
5. Between $36,000 and $45,000
6. Between $45,000 and $60,000
7. Between $61,000 and $75,000
8. Between $76,000 and $100,000

9. More than $100,000

Q9. Marital status
1. Single
2. Couple/free union
3. Married
4. Divorced
5. Widowed

Q10. Education
1. Did not complete secondary school
2. Finished high school
3. Some university or vocational certificate
4. Associate's degree
5. Baccalaureate (university) degree
6. Advanced degree (Master's, MD, DDS, PhD, etc.)

Q11. How many children do you have?
1. 1
2. 2
3. 3
4. 4
5. 5 or more

Q12. Most of my family lives...
1. In the United States
2. In my home country
3. In a country other than the United States or my home country

GENERAL VACCINE ATTITUDES
Please indicate your level of agreement with each of these statements.
Scale: (1) Strongly Disagree
(2) Disagree
(3) Neither Agree nor Disagree
(4) Agree
(5) Strongly Agree

Q1. Vaccines are more beneficial than harmful.
Q2. Vaccines are effective in preventing diseases.
Q3. Vaccines are extensively evaluated to ensure their safety.
Q4. My children have received all their recommended vaccines.
Q5. Vaccination efforts have reduced infectious diseases in the United States.

HPV VACCINE UPTAKE
Q1. Have you been vaccinated against HPV?
1. Yes
2. No

3. Don’t know

Q2. Which of the following best describes your attitude about giving the HPV vaccine to your children?

1. I will not vaccinate my children
2. I probably won't vaccinate my children
3. I am thinking about vaccinating my children, but I haven't made a decision yet
4. I plan to vaccinate my children
5. I have already vaccinated my children

Please indicate your level of agreement with each of these statements.
Scale: (1) Strongly Disagree
(2) Disagree
(3) Neither Agree nor Disagree
(4) Agree
(5) Strongly Agree

Q3. I would recommend the HPV vaccine to my neighbors and friends.
Q4. I would encourage my family to get vaccinated and to vaccinate their children.
Q5. I am in favor of my family getting the HPV vaccine.

ATTITUDES TOWARD HPV VACCINE

Please indicate your level of agreement with each of these statements.
Scale: (1) Strongly Disagree
(2) Disagree
(3) Neither Agree nor Disagree
(4) Agree
(5) Strongly Agree

Q1. I believe it is safe to give the HPV vaccine to my children.
Q2. I believe that the HPV vaccine is a necessary preventive measure for my children.
Q3. I think the vaccine is designed to protect my children.
Q4. I think the vaccine is more helpful than harmful.

KNOWLEDGE OF HPV

Please indicate your level of agreement with each of these statements.
Scale: (1) Strongly Disagree
(2) Disagree
(3) Neither Agree nor Disagree
(4) Agree
(5) Strongly Agree

Q1. HPV is a potentially life-threatening infection.
Q2. HPV infection can cause severe physical suffering.
Q3. Only a small minority of people will get HPV in their lifetime.
Q4. HPV causes a substantial amount of cancer in women and men.

OPENNESS/COMFORT/FEAR

Q1. I would like to learn more about the HPV vaccine.

1. Strongly disagree
2. Disagree
3. Neither agree nor disagree
4. Agree
5. Strongly Agree

Q2. I might consider vaccinating my children if I learn more about it.

1. Strongly disagree
2. Disagree
3. Neither agree nor disagree
4. Agree
5. Strongly Agree

Q3. I am afraid of the vaccine

1. Strongly disagree
2. Disagree
3. Neither agree nor disagree
4. Agree
5. Strongly agree

Q4. I am comfortable deciding about the HPV vaccine

1. Strongly disagree
2. Disagree
3. Neither agree nor disagree
4. Agree
5. Strongly Agree

ENGLISH PROFICIENCY

Please indicate your level of agreement with each of these statements.
Scale: (1) Not at all comfortable
(2) Not very comfortable
(3) Neither comfortable nor uncomfortable
(4) Most comfortable
(5) Totally comfortable
(6) Not applicable

Q1. How comfortable do you feel speaking English at work?
Q2. How comfortable do you feel speaking English at home?
Q3. How comfortable are you speaking English in public?
Q4. How comfortable are you with understanding English?

Intervention Script

Instructions for main video

Watch this video about HPV. It is a 5-minute compilation of short clips taken from longer videos that you can watch if you wish. (Note: You will not be able to move on to the next question until you have watched the whole video).

Additional videos
Would you like to watch additional videos to know more? Please choose one of the options below.
1. I would like to know more about the HPV virus.
2. I would like to know more about how to prevent HPV.
3. I would like to know more about the development of the HPV vaccine.
4. I would like to know more about why the HPV vaccine is recommended for boys.
5. I would like to know more about the side effects of the HPV vaccine.
6. I would like to see testimonials from parents who have decided to vaccinate their children.
7. I don't want to see any more videos

[Skip To: End of Block If you want to know more? Choose one of the following options = I don't want to see any more videos.]

[Additional videos appeared based on selection, followed by one question]

Follow-up Question
How would you rate this video
1. Excellent
2. Good
3. Fair
4. Poor

Post-Intervention Questions

GENERAL VACCINE ATTITUDES
(Same as above)

VACCINE UPTAKE
(Same as above)

ATTITUDES TOWARD HPV VACCINE
(Same as above)

KNOWLEDGE OF HPV
(Same as above)

OPENNESS/COMFORT/FEAR
(Same as above)

CARE LEVEL
Please indicate your level of agreement with each of these statements.
Scale: (1) Strongly Disagree
(2) Disagree
(3) Neither Agree nor Disagree

(4) Agree
(5) Strongly Agree

Q1. The makers of these videos have my best interests at heart.
Q2. I feel that a significant effort was made to ensure that I understood this material.
Q3. Having these videos in Spanish makes me feel that I am being more cared for.
Q4. Having these videos in Spanish makes me feel like I am part of the conversation.
Q5. These videos were made in a way that responded to my specific needs.

VIDEO SPECIFIC FEEDBACK

Please indicate your level of agreement with each of these statements.
Scale: (1) Strongly Disagree
(2) Disagree
(3) Neither Agree nor Disagree
(4) Agree
(5) Strongly Agree

Q1. I feel that these videos provided reliable information about the HPV vaccine.
Q2. These videos were easy to understand.
Q3. These videos were enjoyable.
Q4. I feel more informed about the HPV vaccine because of these videos.
Q5. I liked the way the videos were presented.
Q6. I would like to see more videos like these on other vaccines and health interventions.
Q7. These videos helped me change my mind about allowing HPV vaccination for my children.
Q8. These videos helped me decide about allowing HPV vaccination for my children.
Q9. These videos confirmed my decision to allow HPV vaccination for my children.
Q10. I would recommend these videos to others.

Software
Sonix transcription software
Version- Premium version.
Source-Sonix.ai
Date used

- Focus group one- February 13th 2024
- Focus group two- March 4th 2024

DeepL translator
Version- Pro Starter.
Source- DeepL GmbH
Date used

- Focus group one- March 10th 2024
- Focus group two- April 5th 2024

Mplus

Version- 8.
